# Non-conventional superconductivity in magnetic In and Sn nanoparticles

**DOI:** 10.1038/s41598-022-04889-6

**Published:** 2022-01-14

**Authors:** Ma-Hsuan Ma, Erdembayalag Batsaikhan, Huang-Nan Chen, Ting-Yang Chen, Chi-Hung Lee, Wen-Hsien Li, Chun-Ming Wu, Chin-Wei Wang

**Affiliations:** 1 Department of Physics, National Central University, Zhongli, 32001 Taiwan; 2 National Synchrotron Radiation Research Center, Hsinchu, 30076 Taiwan

**Keywords:** Nanoscience and technology, Physics

## Abstract

We report on experimental evidence of non-conversional pairing in In and Sn nanoparticle assemblies. Spontaneous magnetizations are observed, through extremely weak-field magnetization and neutron-diffraction measurements, to develop when the nanoparticles enter the superconducting state. The superconducting transition temperature T_C_ shifts to a noticeably higher temperature when an external magnetic field or magnetic Ni nanoparticles are introduced into the vicinity of the superconducting In or Sn nanoparticles. There is a critical magnetic field and a critical Ni composition that must be reached before the magnetic environment will suppress the superconductivity. The observations may be understood when assuming development of spin-parallel superconducting pairs on the surfaces and spin-antiparallel superconducting pairs in the core of the nanoparticles.

## Introduction

Phonon-mediated *s*-wave pairing between the electrons near the Fermi level forms spin-singlet (S = 0) Cooper pairs. This pairing has become the backbone of BCS superconductivity. The BSC mechanism explains most, if not all, of the physical properties associated with the so-called conventional weak-coupling superconductor. In this context, the elements In and Sn, in their bulk form, behave as a standard BCS-type superconductor, where the magnetic environment will destroy the spin-singlet pairings. In principle, Cooper pairs can also form in other symmetries, such as the spin-triplet *p*-wave^1,2^, or can be mediated through other quasi-particles, such as spin fluctuations^3–5^. Spin-triplet *p*-wave superconductivity has been identified in the heavy-fermion compound UPt_3_^6,7^ as well as in the quasi-two-dimensional ruthenate Sr_2_RuO_4_^1,8^. Spin-singlet *d*-wave pairing has been found in the high-T_C_ cuprate YBa_2_Cu_3_O_7_^9^. Cooper-pair moments can develop in the superconducting state that is associated with a spin-triplet pairing, as has been observed in Sr_2_RuO_4_ by polarized neutron diffraction studies^10,11^. Although the superconductivity of elements in their bulk form is believed to be associated with the spin-singlet *s*-wave pairing, it is now known that superconducting parameters depend strongly on the physical size of the system^12–23^. Although the most noticeable finite size effect is the loss of superconductivity upon reaching the Anderson criterion^24–26^ when the electron level separation near the Fermi level becomes comparable to the BCS superconducting gap. There is, however, a particular range in particle size which reveals nonconventional superconductivity prior to entering the Anderson regime. Our previous results reveal noticeable enhancement of the superconducting transition temperature T_C_ and critical magnetic field H_C_ in extremely space-restricted Pb^14,15^, In^17^, Sn^22^ and Al^23^ nanoparticles before reaching the Anderson regime. Furthermore, the superconductivity which coexists with ferromagnetism at low temperatures^22,23^ can be attributed to the enhanced superconductivity that survives from the local spin polarized ferromagnetic moments developed in the nanoparticles.

In searching for superconductivity in quantum sized nanoparticles from other than BCS pairings, we study the effects of an external magnetic field or magnetic proximity on the superconductivity in extremely space-restricted In and Sn nanoparticles. Here, we report on the results of magnetization, magnetic susceptibility, resistivity and neutron diffraction measurements made on In, Sn and In/Ni nanoparticle assemblies. Development of additional magnetization in the superconducting state is revealed. The existence of an intrinsic magnetic moment in the superconducting state is confirmed by the neutron diffraction measurements. An enhancement of superconductivity by the application of an external magnetic field was observed, with the enhancement in T_C_ becoming even larger with the introduction of magnetic Ni nanoparticles into the nanoparticle assembly. An inverse magnetic proximity effect was also observed. T_C_ of the superconducting nanoparticles increases noticeably, when magnetic Ni nanoparticles are introduced into the vicinity of the superconducting nanoparticles. These effects are then reversed when the external magnetic field reaches a critical strength or when the concentration of the neighboring magnetic Ni nanoparticles reaches a critical composition. These results indicate the appearance of non-conventional coupling for the superconductivity of In and Sn nanoparticles.

## Materials and methods

### Synthesis of nanoparticles

Two sets of In (designated as In-A and In-B), one of Sn (designated as Sn-A), and one of Ni (designated as Ni-A) nanoparticles were fabricated employing the gas-condensation method, following the steps taken in Ref.^14^. High-purity (99.99%) In/Sn/Ni spheres (2–2.5 mm in diameter) were evaporated in an Ar atmosphere at selective pressures (Table 1), using an evaporation rate of 0.05 Å/s. The evaporated particles were collected on a non-magnetic plate, which was placed 20 cm above the evaporation source and the temperature was maintained at 77 K. After restoration to room temperature, the nanoparticles, which were only loosely attached to the collector, were stripped off from the collector plate. The samples thus obtained were in powdered form and consisted of a macroscopic amount of individual In/Sn/Ni nanoparticles. There were no substrates or capping molecules on these nanoparticles. The nanoparticles were kept in a vacuum at all times, expect when being mixed together before being loading into the sample holders. This was done in an Ar atmosphere and took less than 5 min.

**Table 1:** Chamber pressures used during evaporation, mean particle diameters, standard deviation widths of the size distributions, saturation magnetizations at 300 K, and labels used for the nanoparticle assemblies used in this study.

Element	P (torr)	<d> (nm)	σ	M_S_ (emu/g)	Label
In	2.0	7.0 (2)	0.11 (1)	0.126 (4)	In-A
In	1.0	10.6 (4)	0.12 (1)	0.105 (5)	In-B
Sn	1.5	7.0 (4)	0.18 (4)	0.279 (3)	Sn-A
Ni	4.5	4.5 (3)	0.11 (2)	28.0 (2)	Ni-A

P = Chamber pressure used during evaporation.

<d> = Mean particle diameter.

σ = Standard deviation width of size distribution.

M_S_ = Saturation magnetization at 300 K.

### Methods

The nanoparticle (NP) assembly was obtained by thoroughly mixing nanoparticles A and B with a mass ratio of A:B = m:n, hereafter designated as (A)_m_(B)_n_. For example, (In-A)_90_(Ni-A)_10_ indicates that in this sample, the mass ration of In-A:Ni-A = 90:10. After mixing, the powder was shaken at 10 Hz for 3 min using a Vortex-Genie Mixer. Packing fraction of $f\equiv \frac{\mathrm{mass} \, \mathrm{density} \, \mathrm{ of} \,  \mathrm{the} \, \mathrm{ nanoparticle} \, \mathrm{assembly}}{\mathrm{mass}\,  \mathrm{density}\,  \mathrm{of} \,  \mathrm{its} \,  \mathrm{counterpart} \,  \mathrm{ in} \,   \mathrm{bulk} \, \mathrm{form}}\times 100\%$ is used to specify the mean separation between nanoparticles in the assembly.

The x-ray diffraction measurements were conducted using a Bruker D8 ADVANCE diffractometer with an incident wavelength of λ = 1.5406 Å from a copper target, a Bruker LynxEye linear position sensitive detector (PSD) captured a scattering angle of 4°, and a Ni filter was placed before the PSD to screen the *K*_β_ radiation. The diffraction patterns were taken in the reflection geometry. The neutron diffraction measurements were conducted at the Bragg Institute, ANSTO, using the high intensity powder diffractometer Wombat, employing Ge(113) monochromator crystals to select an incident wavelength of λ = 2.412 Å and a cylindrical vanadium-can to hold the nanoparticles (~ 0.7 g). The sample temperature was controlled using a He-gas closed-cycle refrigeration system.

Magnetization, ac magnetic susceptibility, dc electrical resistivity and specific heat measurements were all performed on a Physical Property Measurement System manufactured by Quantum Design, employing the standard setup. For the magnetization and susceptibility measurements, the nanoparticles (~ 70 mg) were packed into a non-magnetic cylindrical holder also from Quantum Design, which produces a smooth temperature curve and background signals which are ~ 4% that of the sample. For the resistivity measurements, samples in the form of solid pieces were obtained by cold-pressing the powder flat using a mechanical pressure of 5–20 kgW/cm^2^ (depending on the designed packing fraction), after thoroughly mixing nanoparticles in the designed mass ratios. The typical sample size was ~ 2 × 2 × 0.1 mm^3^ which could be handled normally. The resistivity data were collected using the standard four-probe setup, operated in constant current mode. The specific-heat data were collected employing the thermal relaxation method, with a charcoal pump placed near the sample platform to avoid He condensation. The nanoparticles were supported using the N-Grease by Apiezon, which produces ~ 5% of the total signal and a smooth temperature curve.

### Sample characterization

**Figure 1:**
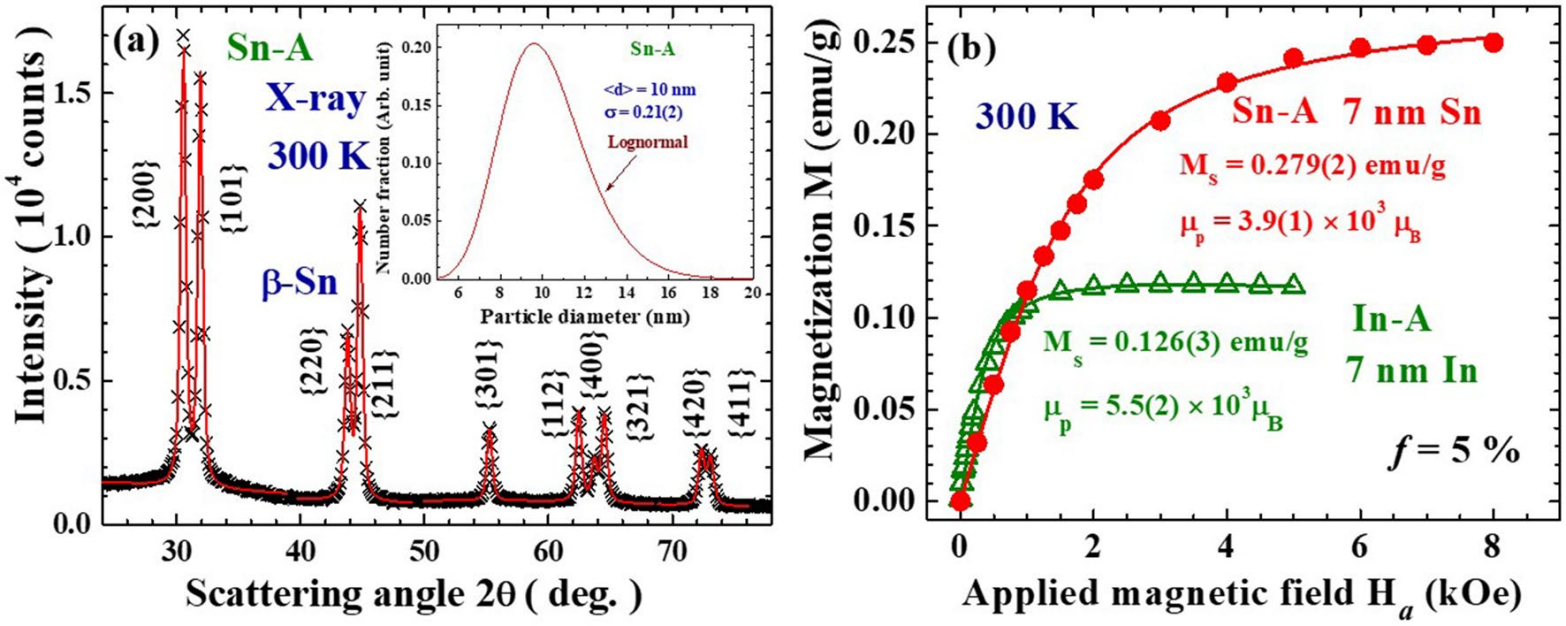
(**a**) X-ray diffraction pattern of the Sn-A nanoparticle assembly, taken at room temperature, revealing a tetragonal β-Sn structure. The solid curves indicate the calculated profile using the size distribution shown in the inset, giving a mean particle diameter of 10 nm for the Sn-A nanoparticles. (**b**) Isothermal magnetization curves of 7 nm In-A (open triangles) and 7 nm Sn-A (filled circles) nanoparticle assemblies at a packing fraction of *f* = 5%, measured in field-increasing loops at 300 K. The solid curves indicate the fits of the data to the Langevin profile, giving saturation magnetizations of 0.126(3) and 0.279(2) emu/g for the 7 nm In-A and 7 nm Sn-A nanoparticles, respectively.

Figure 1a show the X-ray diffraction pattern of the representative Sn-A NP assembly, revealing the NPs crystallize into the same structure as their bulk counterpart. There are no identifiable traces of oxidation phases in the diffraction patterns. As expected, the diffraction peaks appear to be much broader than the instrumental resolution, reflecting the broadening of the peak profiles from the finite-size effect. The mean particle diameter and size distribution of the NP assembly were determined by fitting the diffraction peaks to the diffraction profiles of finite sized particles^27^. The solid curves in Fig. 1a indicate the diffraction pattern calculated assuming a log-normal size distribution (inset to Fig. 1a) with a mean particle diameter of 10.0(3) nm and a standard deviation of 0.21(2) for the Sn-A NP assembly. The chamber pressure used during evaporation, the mean particle diameter and the standard deviation of size distribution for the four sets of NP assemblies are listed in Table 1.

## Results and discussion

### Ferromagnetic spin polarization

The isothermal magnetization curves M(H_*a*_) of the NP assemblies reveal a rapid increase with increasing H_*a*_ in the low H_*a*_ regime, reaching saturation at a higher H_*a*_. Figure 1b shows representative M(H_*a*_) curves of 7 nm In-A (open triangles) and 7 nm Sn-A (solid circles) NP assemblies taken at 300 K. The M(H_*a*_) can be described (solid curves on data) very well by a Langevin profile of M(H_*a*_) = M_S_[coth(x) − (1/x)], where M_S_ is the saturation magnetization, x ≡ μ_P_H_*a*_/k_B_T, μ_p_ is the mean particle moment and k_B_ is the Boltzmann’s constant, giving M_S_ = 0.126(2) and 0.279(2) emu/g for the In-A and Sn-A NP assemblies, respectively, at 300 K. The Langevin behavior of M(H_*a*_) may be understood as the alignment of a randomly oriented assembly of magnetic nanoparticles, each characterized by a superspin with a mean particle moment μ_p_, at a temperature T by the applied magnetic field H_*a*_. Similar Langevin M(H_*a*_) curves were also observed for the 4.5 nm Ni-A NP assembly, giving a M_S_ = 28.0(2) emu/g at 300 K. Note that the M_S_ of bulk Ni is 58.6 emu/g at 300 K. The M_S_ for the four sets of NP at 300 K are listed in Table 1. A larger M_S_ was obtained for a smaller In NPs (Table 1). This reveals that the contribution from the surface spins to particle superspin dominates that from the core spins in In NPs. On the other hand, a smaller M_S_ was obtained for Ni NPs, showing the core spins dominate over the surface spins in Ni NPs.

### Ferromagnetic moment in superconducting state

**Figure 2:**
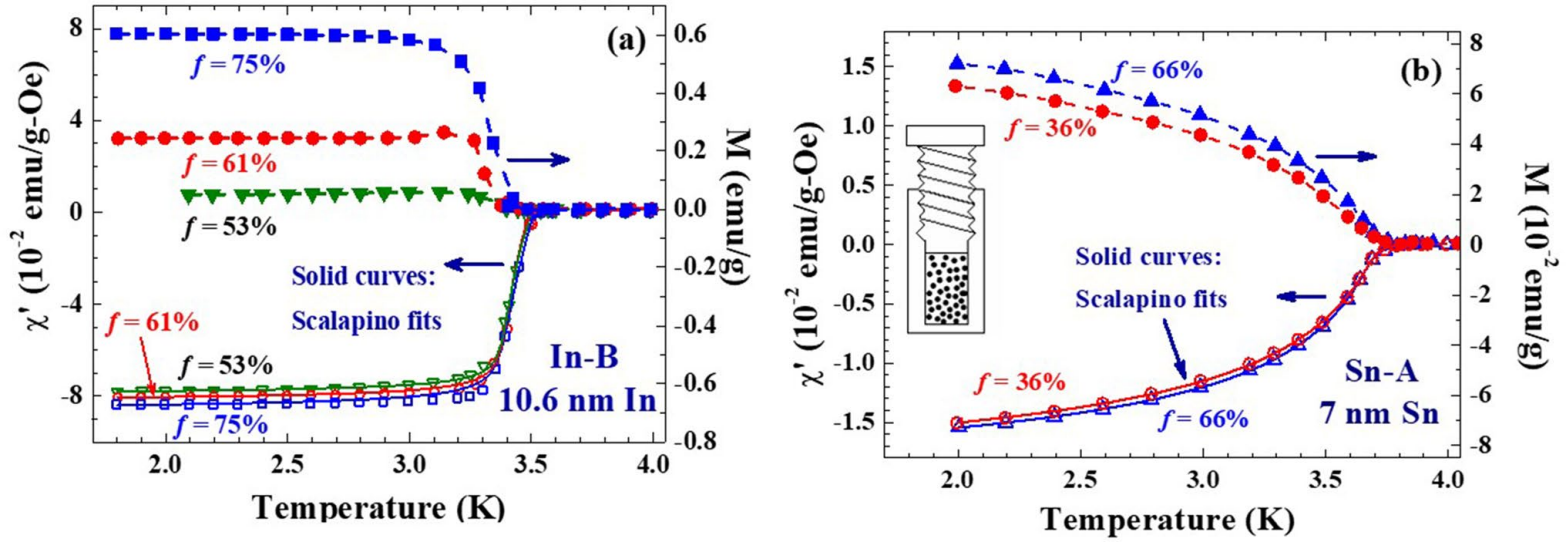
Direct comparison of the M(T) and χ′(T) curves of (**a**) 10.6 nm In and (**b**) 7 nm Sn nanoparticle assemblies, taken at various packing fractions. The solid curves on χ′(T) indicate the fits of the data to Scalapino’s expression for superconducting screening. The dashed curves on M(T) act as a guide to the eye only. The inset to (b) shows a schematic drawing of the device used to adjust the packing fraction of the nanoparticle assembly.

A packing fraction of *f* ≈ 5% is frequently obtained when naturally packs the assembly into a holder. Using the holder shown in the inset to Fig. 2b, the packing fraction can easily be adjusted by turning the tap cap. This set-up allows us to fine tune the packing fraction of the assembly and to perform measurements on the very same nanoparticles at different packing fractions. The highest achievable packing fraction obtained in the present study is *f* = 75%. Figure 2 displays the temperature dependence of the magnetization M and the in-phase component χ′ of the ac magnetic susceptibility, taken at various packing fractions, of the In-B (Fig. 2a) and Sn-A (Fig. 2b) NP assemblies. The magnetizations were measured without the presence of an external magnetic field or a driving magnetic field, except a residual dc magnetic field of ~ 3 Oe that may still appear, but to detect the magnetization induced in the sensing coil when the sample was removed from the coil. This measures the spontaneous magnetic moment of the sample. The χ′, on the other hand, measures the response when the sample is subjected to a weak probing ac magnetic field. This reveals the response of the sample to the probing magnetic field. The diamagnetic χ′ signals the appearance of superconductivity at low temperatures. These χ′(T) can be described (solid curves) by Scalapino’s expression^28^ to give T_C_ = 3.486(3) and 3.714(2) K for In-B at *f* = 53% and Sn-A at *f* = 36%, respectively.

Interestingly, spontaneous magnetizations appear in the superconducting regime. The magnetization begins to develop at a temperature that is slightly but noticeably lower than the development of superconductivity. This component disappears in the normal state. It appears that superconductivity triggers the development of spontaneous magnetization, with the magnetic moment points, in some degree, in the same direction of macroscopic magnetization of the assembly. This is a behavior that will not appear in the superconducting state with a spin-single S = 0 pairing. The M(T) curves measured with an applied magnetic field H_*a*_ exhibit a diamagnetic screening effect, as expected. Increasing packing fraction will progressively enhance the overlaps of the wavefunctions of neighboring particles, leading to the broadening of the conduction band, which in terms lowers the Fermi energy of the surface electrons that causes a portion of the electrons to flow back to the surface region. The larger magnetization observed at a higher packing fraction reflects that closing of interparticle separation gives rise to an increase of the number of uncompensated electrons on the particle surfaces.

**Figure 3:**
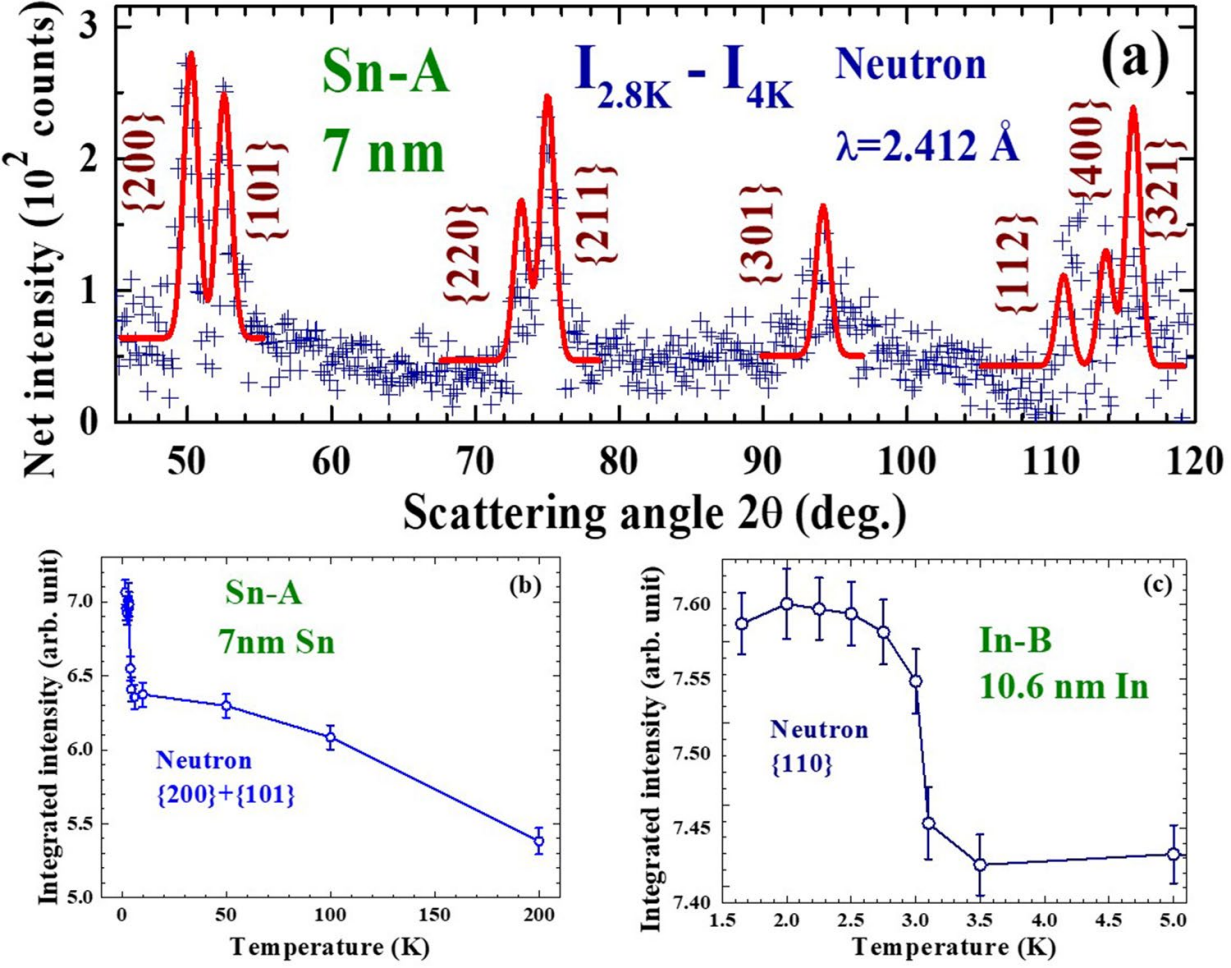
(**a**) Difference pattern of the 7 nm Sn nanoparticles between the neutron diffraction patterns taken at 2.8 and 4 K, revealing significant enhancement of the reflection intensities in the superconducting state. (**b**) Temperature dependence of the (200) + (101) reflection of the 7 nm Sn nanoparticles, revealing a ~ 19% increase in the intensity upon cooling from 200 to 4 K and an additional 10% increase upon further cooling to 1.65 K. (**c**) Temperature dependence of the (110) reflection of the 10.6 nm In nanoparticles, revealing a progressive increase in the intensity upon cooling to the superconducting state.

The existence of intrinsic magnetic moments in 7 nm Sn-A and 10.6 nm In-B NP assemblies is confirmed by the neutron diffraction measurements. Increases in the reflection intensities of the 7 nm Sn NPs upon cooling from 4 to 2.8 K are clearly revealed in the difference pattern between the diffraction patterns taken at 2.8 and 4 K (Fig. 3a). These magnetic intensities appear at the positions of the nuclear Bragg reflections, showing the development of a ferromagnetic moment upon cooling from 4 to 2.8 K. The width of the magnetic peak is the same as that of the associated nuclear Bragg reflection, showing that the magnetic moments are distributed throughout the whole nanoparticle, rather than being located solely on the surface. Unfortunately, the difference between the magnetic moments of the ions in the core and on the surface cannot be resolved, if they are indeed different, at the instrumental resolution used in the present study. Order parameter measurement reveals the integrated intensity of the (200) + (101) reflections increases progressive with decreasing temperature, with a sharp change in the increase rate below 4 K (Fig. 3b). In the normal state the (200) + (101) intensity increases by ~ 19% upon cooling from 200 to 4 K, and an additional 10% increase is seen upon entering the superconducting state on further cooling from 4 to 1.65 K. The thermal reduction rates of the magnetic intensities in the normal and superconducting states differ by a factor of 42, showing that they are associated with different origins. The magnetic diffraction pattern shown in Fig. 3a can be described (solid curve) reasonably well by assuming the development of a ferromagnetic moment of <µ_Z_>  = 0.064 µ_B_ that points in the [101] crystallographic direction. The moment developed in the superconducting state upon cooling from 4 to 1.65 K is <µ_Z_>  = 0.046 µ_B_. A similar behavior of sharp increases in the (110) intensity upon entering the superconducting state is also seen in the 10.6 nm In-A NP assembly (Fig. 3c), revealing the development of an additional ferromagnetic moment in the superconducting state for the 10.6 nm In NPs.

### Superconductivity enhanced by an external magnetic field

**Figure 4:**
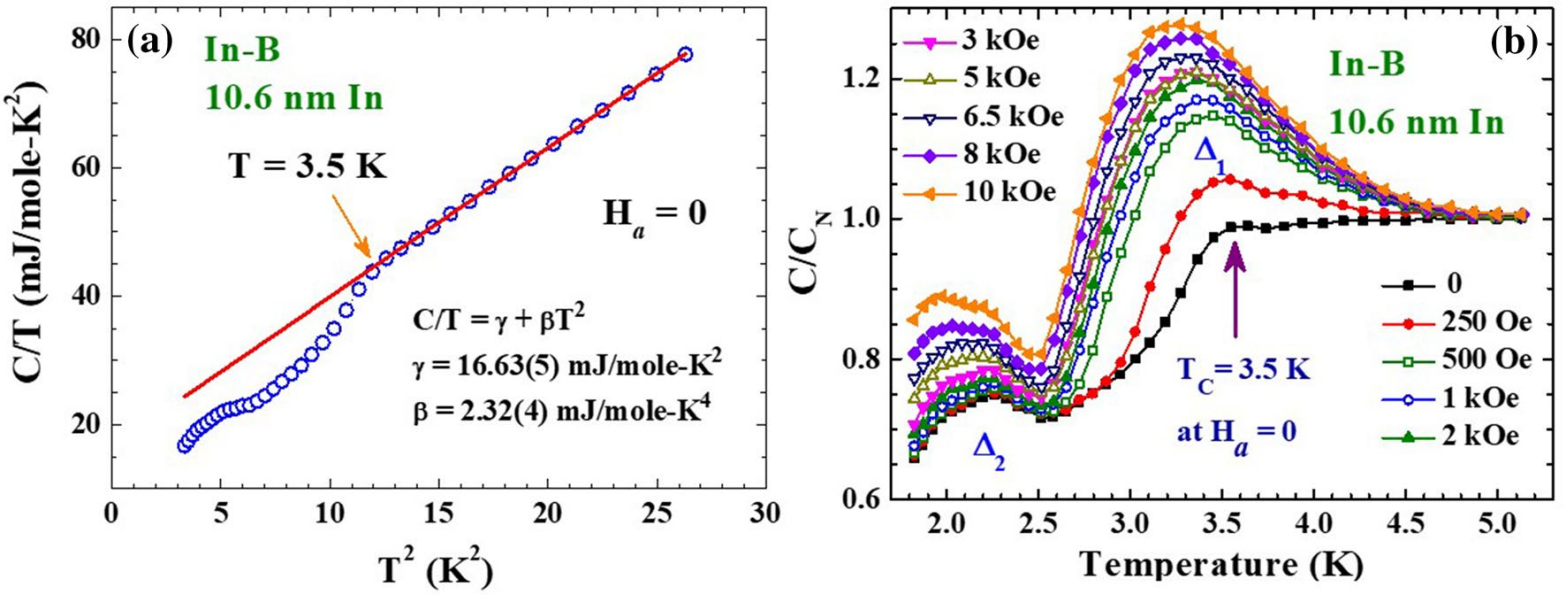
(**a**) C/T vs T^2^ plot of the specific heat of the 10.6 nm In nanoparticles. The solid curve shows the results of the fit of the data at high temperatures to the expression listed in the plot. The specific heat departs from the γT + βT^3^ behavior below 3.5 K. (**b**) Electronic specific heat of the 10.6 nm In nanoparticles measured under various applied magnetic field, revealing a two-peak structure, labelled Δ_1_ and Δ_2_.

The zero-field thermal specific heat of the In-B NP assembly departs greatly from the normal state behavior of γT + βT^3^ upon cooling to below 3.5 K, reflecting the appearance of superconductivity below T_C_ = 3.5 K (Fig. 4a), which is 3% higher than the T_C_ = 3.41 K of bulk In. A lattice coefficient of β = 2.32 mJ/mole-K^2^, corresponding to a Debye temperature of 113 K, is obtained for the 10.6 nm In NPs, showing a reduction of 12% in the Debye temperature upon reduction of the particle diameter to 10.6 nm. Two components, marked Δ_1_ and Δ_2_, that respond differently to H_*a*_ are seen in the electronic specific heat obtained by subtracting the βT^3^ contribution from the data (Fig. 4b). Clearly, Δ_1_ is associated with the occurrence of superconductivity. The application of an H_*a*_ greatly enhances the electronic specific heat in the superconducting transition regime below as well as above T_C_, with the enhancement becoming smaller at a higher H_*a*_. The creation of a spin-polarized gap near the Fermi level by the H_*a*_ cannot account for the observed characteristic H_*a*_-dependence of Δ_1_, since a larger H_*a*_ will generate a larger spin-polarized gap. It clearly shows that the application of an H_*a*_ will alter the electronic behavior in the superconducting state. The Δ_2_ that appears at 2.2 K is less sensitive to the H_*a*_. It is linked to the emergence of the discrete electron level, known as the Kubo gap, near the Fermi level in the 10.6 nm In NPs. A Kubo gap of 0.18 meV is expected for the 10.6 nm In NPs, when is estimated according to the Kubo formula^29–31^. This energy gap, which corresponds to a thermal energy of 2.1 K, agrees with the thermal position of Δ_2_.

**Figure 5:**
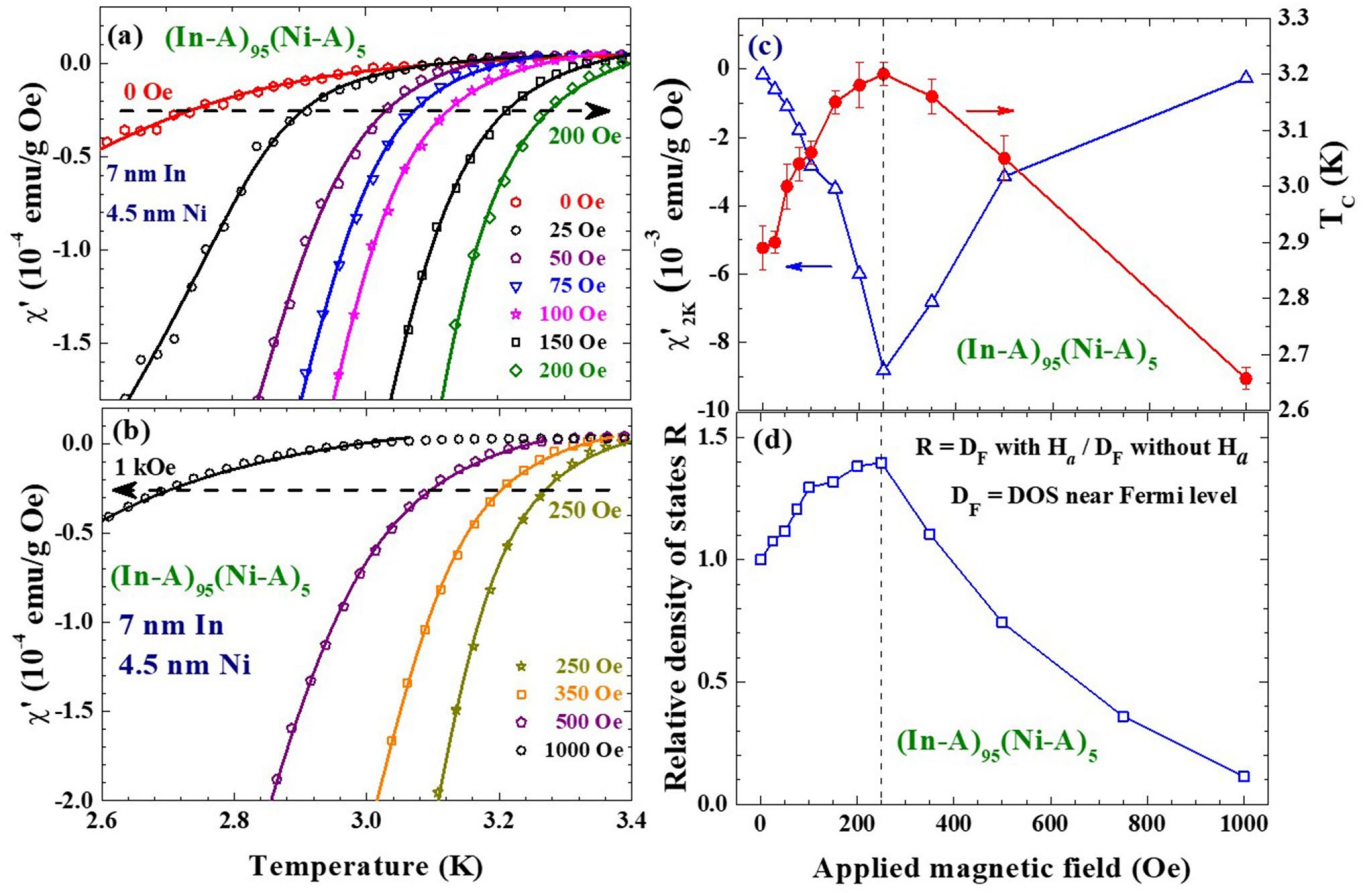
(**a**, **b**) Effects of the applied magnetic field on the χ′(T) curves of (In-A)_95_(Ni-A)_5_ nanoparticle assembly, revealing alterations of T_C_ as the applied magnetic field is changed. The solid curves indicate the results of the fits of the data to Scalapino’s expression for superconducting screening. (**c**) Variations of the value of χ′ at 2 K (open triangles) and T_C_ (filled circles) with the applied magnetic field of (In-A)_95_(Ni-A)_5_ nanoparticle assembly, revealing a critical magnetic field for maximum T_C_ and diamagnetic response χ′_2 K_. (**d**) Variation of the relative density of states near the Fermi level R with an applied magnetic field of (In-A)_95_(Ni-A)_5_ nanoparticle assembly, revealing a critical magnetic field for maximum R.

**Figure 6:**
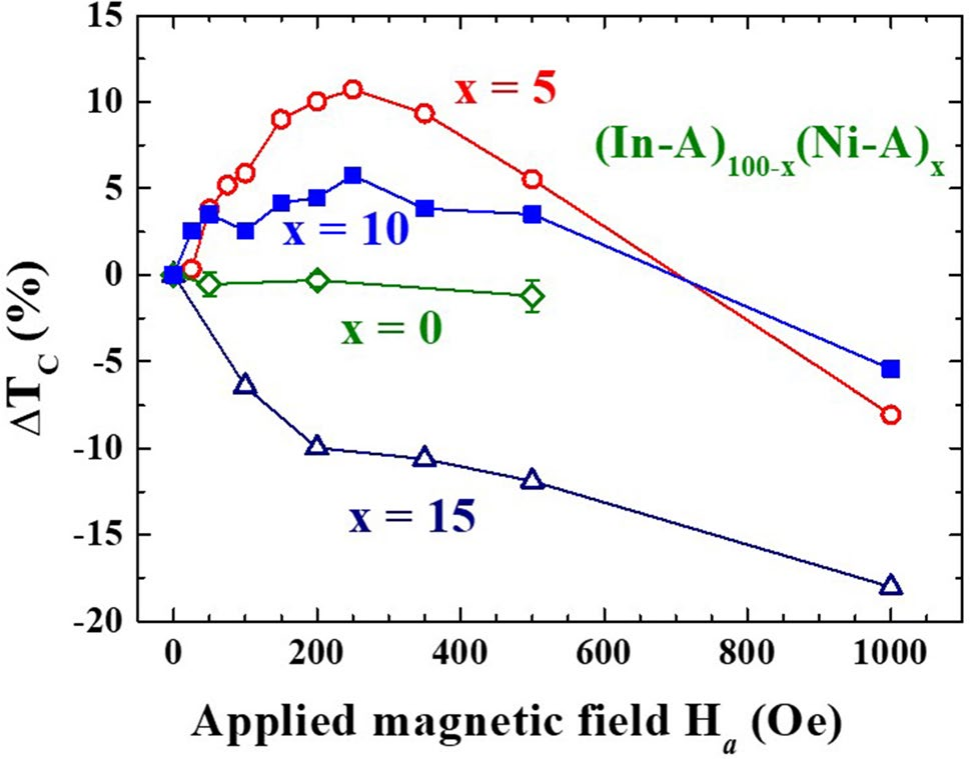
Changes of T_C_ with applied magnetic field of the (In-A)_100−x_(Ni-A)_x_ nanoparticle assemblies at x = 0 (open diamonds), x = 5 (open circles), x = 10 (filled squares), and x = 15 (open triangles), revealing the appearance of a critical applied magnetic field for maximum change of T_C_ in the x = 5 and 10 curves.

The enhancement of T_C_ by the application of a magnetic field H_*a*_ is seen in the (In-A)_100−x_(Ni-A)_x_ NP assemblies (Fig. 5), where the measurements were performed on naturally packed assembly of *f* ~ 5%. The average interparticle separation is ~2.7 times the particle diameter at *f* ~ 5%, such that the interparticle interaction is insignificant. T_C_ of (In-A)_95_(Ni-A)_5_ increases progressively as H_*a*_ increases from 0 to 250 Oe (Fig. 5a), but then decreases with a further increase in H_*a*_ (Fig. 5b). In addition, the diamagnetic response, represented by the value of χ′ at 2 K χ′_2K_, is stronger as H_*a*_ increases from 0 to 250 K, but becomes weaker upon a further increase in H_*a*_ (open triangles in Fig. 5c). The χ′(T) can be described by Scalapino’s expression (solid curves in Fig. 5a,b) used to extract T_C_ together with the density of states (DOS) near the Fermi level D_F_^28^. T_C_ of the 7 nm In NPs increases from 2.89 K at H_*a*_ = 0 to 3.21 K at H_*a*_ = 250 Oe. The 11% increase of T_C_ by an H_*a*_ of 250 Oe is accompanied by a 40% increase of D_F_ (Fig. 5d). T_C_, D_F_ and χ′_2K_ reach their maxima at H_*a*_ = 250 Oe, above which these superconducting parameters are gradually suppressed by the increase of H_*a*_. Apparently, it is the increase of the DOS near the Fermi level by the application of a magnetic field that strengthen the superconductivity in the 7 nm In NPs. The enhancement of T_C_ by an H_*a*_ is also seen in the (In-A)_90_(Ni-A)_10_ NP assembly, but T_C_ is suppressed by an H_*a*_ in the 15% Ni-A NP assembly of (In-A)_85_(Ni-A)_15_, and no obvious change of T_C_ with H_*a*_ up to 500 Oe is seen in the pure In-A NP assembly (Fig. 6). An inhomogeneous magnetic environment for the In NPs that is generated by the magnetic Ni NPs is apparently essential to reveal the enhancement. Clearly, there are two competing factors at work, one enhancing while the other suppresses the superconductivity which affect the superconductivity in the 7 nm In NPs under an applied magnetic field.

## Conclusions

The superconductivity that operates in the present In and Sn nanoparticles is different in nature from that which operates in bulk In and Sn. The inverse magnetic proximity effect observed in quench-condensed Pb/Ag films^32^ that originated from the leakage of conduction electrons from the Ag to the neighboring Pb films will not appear in the present In/Ni NP assemblies, since the conduction electron density of Ni nanoparticles is significantly lower than that of In nanoparticles. It has been demonstrated^33^ that superconducting properties in a two-dimensional granular superconductor-insulator matrix can be altered by an external magnetic field through the change of the fraction of the superconductor and insulator grains. This type of coupling is unlikely to be the main mechanism operated in the present In/Ni nanoparticle assemblies, since it is difficult to understand a magnetic field as weak as 250 Oe can alter the superconductor-insulator fraction large enough in a 5% Ni assembly for a 17% increase of T_C_ in In. It is very unlikely that an external magnetic field as weak as 300 Oe can cause a 5% softening in phonon frequencies to account for the 17% increase in T_C_. It has been theoretically demonstrated that polarization of magnetic impurity spins by a parallel magnetic field can enhance T_C_ in highly disordered thin films^34^. This is unlikely to be the main source for the enhanced T_C_ observed in present In/Ni NP assemblies, since it is difficult to understand the aligned magnetic impurity spins by an H_*a*_ of 250 Oe will drive the DOS to increase by 45% (Fig. 5d) and the strength of magnetic impurity, if they do exist, cannot be stronger than the additional intrinsic ferromagnetic moments that developed in the superconducting state (Fig. 3). A pairing mechanism that can be enhanced by the magnetic field is indeed needed to understand the present observations of T_C_ can be enhanced by an external magnetic field or by magnetic neighbors. The spin-triplet *p*-wave pairing which has been observed in Sr_2_RuO_4_ and UPt_3_ could be a candidate. The observation that superconductivity is eventually suppressed once the external magnetic field or the neighboring magnetic content exceeds a critical composition, showing that there is a superconducting component that can be suppressed by magnetic proximity. The quantum confinement is not yet significant in the present nanoparticles, showing that the surface atoms play a key role. One possible configuration that can be used to understand the present observations is that the antiparallel spin pairings develop mainly at the core, while the parallel spin pairings appear mainly on the surface. In this configuration, an external magnetic field would help with the formation of parallel spin pairings on the surface, but suppress antiparallel spin pairings in the core. Below the critical magnetic field the effect from the surface dominates to enhance superconductivity. Above this point, the magnetic field suppresses superconductivity when the effect from the core dominated. No experimental evidence resulted from this study that may be used to draw a conclusive argument on the pairing mediators for the superconductivity, but to conclude that the enhanced surface effects and proximity effects play an essential role in revealing the non-conventional behavior that exist in In and Sn. The surface effect and proximity effect are both enhanced by reducing the particle size into nano-scale for a higher fraction of atom on the surface and a higher inhomogeneity in local magnetic environment. Finally, we remark that there is no evidence in this study that pairing mechanism could be altered by reducing size into nano-scale.
